# Action Feedback Enables Novices to Implicitly Acquire Task Regularities from Experts During Joint Statistical Learning

**DOI:** 10.3390/bs16071152

**Published:** 2026-07-09

**Authors:** Zheng Zheng, Nanye Deng, Caiyue Yin, Weijian Li, Jun Wang

**Affiliations:** 1 School of Psychology, Zhejiang Normal University, Jinhua 321004, China; zhengz@zjnu.edu.cn (Z.Z.); 13251094467@163.com (N.D.);; 2 Zhejiang Philosophy and Social Science Laboratory for the Mental Health and Crisis Intervention of Children and Adolescents, Zhejiang Normal University, Jinhua 321004, China

**Keywords:** joint action, joint statistical learning, expert-novice, action feedback

## Abstract

Joint statistical learning enables interacting individuals to form shared representations, but prior research has primarily focused on homogeneous dyads with equivalent expertise. Real-world interactions often involve knowledge asymmetries, yet it remains unclear how novices implicitly acquire statistical regularities from expert partners via sensorimotor signals. This study investigated whether novices can implicitly extract sequence regularities to enhance joint statistical learning and compared two candidate mechanisms, action feedback versus action visibility. Using a modified serial reaction time task across three experiments, we found that novices paired with trained experts exhibited significantly steeper declines in reaction time compared to those paired with pseudo-experts. Moreover, expert-paired novices demonstrated a pronounced quadratic trajectory, indicating sequence-specific learning. Experiment 2 revealed that the absence of immediate action feedback eliminated this sequence-specific interference effect in novices, highlighting the critical role of shared perceptual outcomes in the implicit transmission of task regularities. Conversely, Experiment 3 showed that the absence of visual access to the expert’s physical movements attenuated neither the novices’ general sequence acquisition nor their sequence-specific interference effect. These findings extend the Theory of Event Coding framework to asymmetric social contexts by demonstrating that effect-based coding, rather than direct kinematic observation, drives implicit behavioral facilitation in joint action.

## 1. Introduction

Human behavior frequently involves joint actions, ranging from basic attaining interpersonal coordination during a shared walk to complex coordination in a musical duet. The success of such interpersonal coordination depends fundamentally on the ability of co-actors to anticipate and dynamically adapt to one another’s movements. A primary cognitive mechanism driving this predictive adaptation is statistical learning—specifically, the capacity to implicitly extract and internalize environmental regularities to facilitate motor execution (Saffran et al., 1996; Tal & Vakil, 2020; Vékony et al., 2022). In interpersonal contexts, this capacity enables interacting individuals to form shared statistical representations, a phenomenon we refer to as joint statistical learning (Zheng & Wang, 2023, 2024a). Although recent studies have demonstrated this process, their paradigms have relied exclusively on homogeneous dyads (i.e., partners with identical knowledge), leaving a critical, unresolved research gap regarding how joint statistical learning unfolds in epistemically asymmetric pairings. Real-world collaborative interactions often involve knowledge asymmetries—such as a novice coordinating with an experienced partner—where the transmission of regularities proceed implicitly, without explicit verbal instruction. Crucially, it remains unknown whether and how novices can extract underlying sequence-related task regularities from a more knowledgeable partner via the nonverbal sensorimotor cues generated during joint action (Curioni et al., 2019; Loehr & Vesper, 2016; Vesper & Richardson, 2014). A key form of statistical learning is sequence learning, which involves the acquisition of embedded rules within motor skills (Dirnberger & Novak-Knollmueller, 2013; Marsolek & Field, 1999; Tal & Vakil, 2020). Our previous work extended this line of research to joint action contexts, demonstrating that joint statistical learning entails representing and integrating a partner’s task regularity information (Zheng & Wang, 2023). Crucially, this joint learning relies on self-other integration—a cognitive process in which individuals combine complementary task regularities into shared representations (Zheng & Wang, 2024a). Foundational research indicates that individuals then use these shared representations to continuously predict a partner’s behavior and monitor their own actions (Bolt & Loehr, 2017; Sebanz et al., 2006; Zheng & Wang, 2024b). Current evidence shows that this integration is optimized when partners share social affiliations (Galinsky et al., 2005; Zhou et al., 2026), perform congruent physical movements (Feng et al., 2020; Novembre et al., 2014; Varlet et al., 2020), or generate predictable action sequences (van der Weiden et al., 2022).

In classic individual sequence learning tasks, expertise is characterized by two distinct behavioral signatures. First, experts exhibit a typical learning curve characterized by an initial rapid decrease in reaction time, followed by a decelerating phase (Marcus et al., 2006; Song et al., 2008). Second, they show a pronounced interference effect, marked by a rebound in reaction time when random stimuli violate the acquired structural regularities (Tal et al., 2021; Tal & Vakil, 2020; Vakil et al., 2017). When these robust behavioral signatures are transferred to interactive settings, interacting with a more knowledgeable partner significantly alters dyadic performance. This asymmetry advantage primarily stems from the dual nature of expertise: experienced co-actors not only provide highly stable behavioral inputs (Le Runigo et al., 2005; Mallek et al., 2017) but also demonstrate a remarkable capacity for dynamic calibration by flexibly adjusting their predictions to accommodate a novice’s suboptimal timing, thereby offering a critical temporal scaffold (van Nooijen et al., 2025; Wolf & Knoblich, 2022; Wolf et al., 2018). Consequently, the distinct behavioral profile of an expert partner acts as a driver of self-other integration, shaping how novices extract and internalize shared statistical regularities (Tufft & Richardson, 2026; van Nooijen et al., 2024). While sequence learning in the SRT task fundamentally involves extracting spatial regularities (i.e., the specific order of target locations), the joint execution of this task introduces a critical temporal dimension. When interacting with an experienced partner, novices are exposed to this highly stable behavioral input and predictable rhythm. We hypothesize that this predictable rhythm reduces temporal uncertainty, freeing up the novice’s cognitive resources to actively track the spatial locations of the partner’s responses while facilitating the spatiotemporal binding of these events.

To understand exactly how this spatial-temporal integration is physically grounded, it is crucial to examine the specific nonverbal sensorimotor signals transmitted between co-actors. The Theory of Event Coding (TEC; Hommel, 2019) offers one critical framework, positing that actions are primarily encoded through their anticipated perceptual effects rather than their kinematic features. According to TEC’s ideomotor principle, an action and its resulting sensory feedback (e.g., the sound of a keypress) form a unified “event file” within the cognitive system—a coupling demonstrated by the action binding effect, whereby a delayed sensory consequence unconsciously shifts a person’s perceived timing of their own movement (Varga et al., 2024; Wang et al., 2025). Applied to asymmetric interactions, this framework suggests that novices internalize the partner’s statistical regularities by attending to the immediate sensory consequences (i.e., action feedback) of those actions.

An alternative perspective, however, emphasizes direct visual access to physical movements—specifically, the continuous visibility of the moving body part (e.g., the hands)—as the primary mechanism for interpersonal information transfer (Royka et al., 2022). According to theories of sensorimotor communication, action intentions can be inferred from the observable flow of a partner’s hand movements (Donnarumma et al., 2018; Tong et al., 2025). When a novice has visual access to an expert’s hand, the expert may systematically adjust their own kinematic trajectories—for instance, by exaggerating spatial curvature—to implicitly convey task-relevant information (McEllin et al., 2018). Occlusion paradigms have shown that when a partner’s hand is not visible, individuals tend to adjust their kinematic planning (e.g., slowing down or amplifying gestures) to compensate for the loss of visual input (Obhi & Sebanz, 2011; Vesper et al., 2016a; Vesper et al., 2016b). From this viewpoint, novices integrate an expert’s nonverbal signals by observing the expert’s movement kinematics rather than through the sensory outcomes those actions produce (Lokesh et al., 2022; Pezzulo et al., 2019; Vesper et al., 2017). Given that action feedback and action visibility yield contrasting predictions regarding how an expert facilitates joint statistical learning, it is critical to identify which pathway fundamentally underlies novices’ integration of expert signals.

In summary, the current study had two primary aims. First, we examined whether novices can integrate an expert’s nonverbal sensorimotor signals to enhance their own joint statistical learning. Second, we compared the two candidate mechanisms—action feedback versus action visibility—to uncover which pathway fundamentally supports this integration process. To this end, we employed a complementary joint statistical learning paradigm (Zheng & Wang, 2024a), in which a modified serial reaction time task implicitly induced self-other integration in a joint action setup. The experiment featured a learning phase (repeated fixed sequence) and a modulating phase (temporary random sequence). Disrupting the acquired rules causes an interference spike in reaction time, followed by a performance recovery. This generates a distinct quadratic trajectory, serving as a behavioral signature of sequence-specific learning. During this joint task, the dyads sat side-by-side and completed a complementary task together. Experiment 1 established the role of partner expertise by pairing naive participants (novices) with either experts (pre-trained on the target deterministic sequence) or pseudo-experts (pre-trained on random sequences). We hypothesized that novices paired with pre-trained experts would exhibit significantly enhanced implicit sequence acquisition and a more pronounced quadratic trajectory (interference effect) compared to those paired with pseudo-experts. Building on this foundation, Experiments 2 and 3 systematically dissected the mechanisms underlying the implicit transmission of task regularities by manipulating the novice’s access to the expert’s nonverbal cues. Specifically, Experiment 2 manipulated the availability of the expert’s action feedback, hypothesizing that removing this feedback would eliminate the novice’s sequence-specific interference effect. Conversely, Experiment 3 manipulated the novice’s perceptual access to the partner’s motor actions (action visibility), hypothesizing that restricting this kinematic access would not alter the sequence-specific interference effect.

## 2. Experiment 1

Experiment 1 sought to determine whether an expert partner facilitates a novice’s joint statistical learning. To address this question, we paired naïve participants (novices) with either trained experts (who had acquired the target sequence) or pseudo-experts (who had been exposed to random sequences). We hypothesized that novices paired with trained experts would exhibit enhanced sequence acquisition and a more pronounced interference effect compared to those paired with pseudo-experts.

### 2.1. Methods

#### 2.1.1. Participants

The sample size was determined based on a prior power analysis using the WebPower package in R (wp.regression, Zhang & Yuan, 2018). Assuming a medium effect size (*f*^2^ = 0.15), an alpha level of 0.05, and a statistical power of 0.95, the analysis indicated a minimum requirement of 106 participants. To ensure a counterbalanced design across conditions, we recruited 112 undergraduate students (*M* = 20.13 years, *SD* = 1.37; all female) from Zhejiang Normal University to form 56 interacting dyads. All participants were right-handed and had normal or corrected-to-normal vision. Prior to testing, written informed consent was obtained from each participant in accordance with the Declaration of Helsinki, and financial compensation was provided. The Research Ethics Committee of Zhejiang Normal University approved all study protocols.

#### 2.1.2. Apparatus and Stimuli

**Figure 1:**
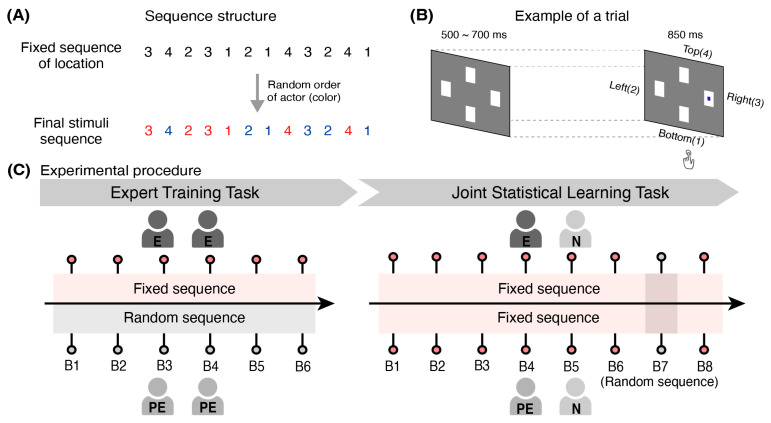
Overview of the procedure of Experiment 1. (**A**) Sequence structure. Target locations followed a fixed 12-item sequential order, with an equal number of red and blue targets (six each); color order was randomized. (**B**) Example of a trial. Temporal organization and spatial layout of an individual trial, showing the four possible target locations (Top, Right, Bottom, Left) and stimulus durations. (**C**) Experimental procedure. Schematic representation of the two main phases: Expert Training Task (Blocks B1–B6) and Joint Statistical Learning Task (Blocks B1–B8; B7 = random sequence block). E = Expert, PE = Pseudo-Expert, N = Novice.

We employed the experimental paradigm and stimulus configurations established in our previous work (Zheng & Wang, 2024a). Stimuli were presented on an LCD monitor (1680 × 1050 pixels; 47 cm × 29 cm) against a gray background using E-Prime 3.0 software. The display consisted of four white squares arranged in a diamond formation corresponding to four spatial locations (up, down, left, and right; approximately 4.6° × 4.6° of visual angle each, see Figure 1A). The target stimulus was a solid square (colored either red or blue; approximately 1.4° × 1.4° of visual angle) that appeared at the center of one of the four white squares. Target locations (coded as 1 = down, 2 = left, 3 = right, and 4 = up) followed one of two distinct 12-item deterministic sequences: Sequence A (e.g., 3-4-2-3-1-2-1-4-3-2-4-1) or Sequence B (e.g., 3-4-1-2-4-3-1-4-2-1-3-2). Within each 12-item sequence, the target appeared in red six times and in blue six times, with the color order pseudo-randomized. To rule out potential confounds arising from specific sequence structures, the target sequences were counterbalanced across dyads regarding their assignment as either the main learning sequence or the interference sequence.

#### 2.1.3. Procedure

The experimental procedure consisted of two consecutive tasks: an Expert Training Task and a subsequent Joint Statistical Learning Task (see Figure 1C). Crucially, naïve participants were randomly assigned to their respective roles (i.e., expert or novice) within each dyad.

Expert Training Task. Two participants jointly performed a sequence learning task to establish distinct levels of implicit sequence familiarity. Each trial began with the presentation of a target (a red or blue square) in one of four spatial locations for a maximum of 850 ms, or until a response was made (see Figure 1B). A 500 ms intertrial interval followed, during which only the four white squares remained on the screen. Participants were instructed to respond to the target location as quickly and accurately as possible using designated keys on a standard keyboard (e.g., arrow keys or the number pad). Their individual responses formed the underlying sequences; to control for spatial and transitional probabilities while preventing explicit recognition, each sequence repetition began at a different ordinal position. The overall training task comprised six consecutive blocks, with each block containing 12 sequence repetitions. Consequently, the training task differentiated participants into two distinct roles: those exposed to the predetermined 12-item sequence were classified as experts, whereas those exposed to the pseudo-random sequence were classified as pseudo-experts.

Joint Statistical Learning Task. This task examined how the partner’s expertise (trained expert vs. pseudo-expert) influenced the implicit statistical learning of a naïve co-actor. To form the experimental dyads, each trained participant was paired with a naïve participant (the novice). The dyads sat side by side in a dedicated testing room to perform a complementary joint sequence task. Crucially, the dyads continuously performed the task across eight consecutive blocks, maintaining the exact trial structures—including timing, color division, and keyboard assignments—established during the training task. To probe sequence-specific learning, Blocks 1 to 6 and Block 8 presented the main sequence, whereas Block 7 introduced an interference sequence. Following the joint task, we assessed participants’ explicit awareness of the sequence structure through a structured debriefing interview. Participants were asked whether they had noticed any underlying patterns or regularities in the stimulus presentations. All participants reported being completely unaware of any such structural regularities, confirming that the statistical learning observed in this study remained implicit.

#### 2.1.4. Data Analysis

Throughout the analysis, Linear Mixed-Effects Model (LMM) analyses were conducted using R (Version 4.2.1; R Core Team, 2022) and the lme4 package (Version 2.0-1) (Bates et al., 2015). To examine whether novices paired with experts and those paired with pseudo-experts exhibited different joint learning dynamics, we calculated the correct reaction times (RTs) and mean error rates separately for each of the conditions (i.e., training condition: expert vs. pseudo-expert, and learning phase: blocks 1–8) as well as for each participant. We first excluded all incorrect responses from the RT analysis. We then excluded participants if their mean error rates were >3 SDs above the overall mean. Accordingly, no participant was excluded because their mean error rate during the learning phase exceeded the predefined threshold. For the remaining participants, specific trials were removed if their RTs were >3 SDs above or below the individual’s block mean. As a result, a total of 410 extreme RT trials, accounting for 1.69% of the total trials, were removed from subsequent analyses. To determine whether error rates warranted separate analysis, we examined the overall accuracy across all experimental blocks. The mean error rate was extremely low (*M* = 0.004, *SD* = 0.009), approaching floor levels. This near-ceiling performance indicates that participants maintained high task compliance and that RT variations were not confounded by speed-accuracy trade-offs. Accordingly, we used RT as the primary dependent variable and did not further analyze error rates.

Following this data screening, the screened RTs were submitted to LMMs to appropriately account for the non-independence and hierarchical structure inherent in our dyadic interaction data. In the models, training condition, learning phase, and their interaction were entered as fixed effects. We verified that all LMMs with the specified nested random-effects structure converged properly and did not yield singular fits. Diagnostic checks of residual Q-Q plots and residual-vs.-fitted plots indicated no substantial violations of normality or homoscedasticity. More importantly, to control for baseline variability, we defined the random-effects structure by including random intercepts for dyad and nested random intercepts for individual participant within the dyad. Fixed effects were tested for significance using Type III likelihood ratio tests via the Anova function from the car package (Version 3.1-5) (Fox & Weisberg, 2019). This approach compares the fit of a full model including the effect of interest against a reduced model without it, yielding a chi-square (χ^2^) statistic that quantifies the improvement in model fit. Effect sizes for the fixed effects were calculated as semi-partial adjusted R^2^ using the r2beta function from the r2glmm package (Version 0.1.3) (Jaeger, 2017). For the expert training task, we modeled training condition (experts vs. pseudo-experts), training phase (Blocks 1 to 6), and their interaction as fixed effects on reaction times, specifying random intercepts to account for variability between dyads and individuals. The joint statistical learning task was analyzed using an identical model structure for the learning phase (Blocks 1 to 6).

To effectively disentangle genuine sequence-specific learning from general practice effects (e.g., motor practice or fatigue effects), we evaluated the modulating phase (Blocks 6, 7, and 8) by contrasting linear and quadratic mixed-effects models. In a classic Sequence–Interference–Recovery design, individuals who have implicitly internalized the sequence regularities demonstrate a characteristic non-monotonic reaction time pattern, driven by the violation of their internal sequence predictions during the interference block (Laubrock & Kinder, 2014; Toh et al., 2021; Verstynen et al., 2012; Zheng & Wang, 2024a). Specifically, performance is highly facilitated during the predictable sequence (Block 6), encounters a transient disruption marked by prolonged reaction times when regularities are violated (Block 7), and recovers immediately upon sequence restoration (Block 8). Because this short–long–short pattern is captured by an inverted U-shaped quadratic curve, a quadratic model provides the optimal framework to isolate the sequence-specific learning effect. Therefore, a superior fit for the quadratic model over the linear model provides a principled statistical test for the presence of true sequence-specific interference.

The linear model was defined as in Equation (1):

RT ~ Phase × Partner Expertise + (1 | Participant | Dyad)(1)

The quadratic model was defined as Equation (2):

RT ~ Phase × Partner Expertise + Partner Expertise × (Phase)^2^ + (1 | Participant | Dyad) (2)

The linear model included modulating phase and partner expertise as fixed effects, whereas the quadratic model added a polynomial term to account for this theoretically expected quadratic trend across the three blocks. Model fits were compared using likelihood ratio tests to determine whether the quadratic model provided a significantly better fit to the modulating phase data than the linear model.

### 2.2. Results

#### 2.2.1. Expert Training Manipulation Validity Test

**Figure 2:**
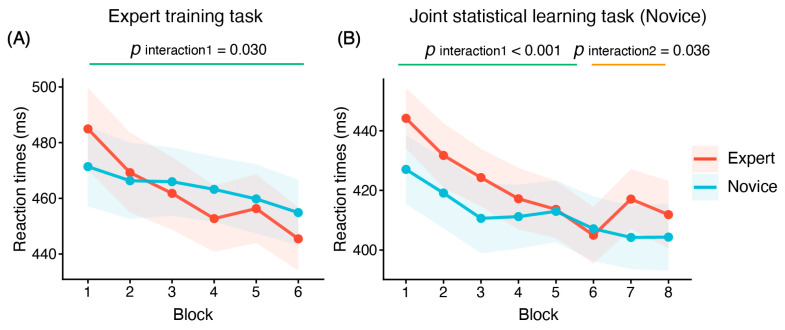
Reaction times across blocks for Expert and Novice groups. (**A**) Expert training task. Mean reaction times (ms) across six training blocks. (**B**) Novice performance in the joint statistical learning task. Mean reaction times (ms) across eight blocks. The green lines denote an interaction between the training conditions and the learning phase (interaction1). The orange line indicates an interaction between partner expertise and the squared phase term during the modulating phase, i.e., Blocks 6–8 (interaction2). Shaded areas represent the 95% confidence intervals.

We first examined whether the trained experts successfully acquired the sequence regularities during the expert training task. The analysis revealed a significant main effect of training condition (*χ*^2^(1) = 28.63, *p* < 0.001, *R*^2^ = 0.09, see Figure 2A) and a significant two-way interaction between training condition and training phase (*χ*^2^(1) = 4.69, *p* = 0.030, *R*^2^ = 0.02). Simple-effect follow-up tests indicated that experts (*χ*^2^(1) = 43.28, *p* < 0.001, *R*^2^ = 0.24) exhibited a significantly steeper rate of reaction time decline compared to pseudo-experts (*χ*^2^(1) = 12.33, *p* < 0.001, *R*^2^ = 0.08).

#### 2.2.2. Novice Performance in the Joint Statistical Learning Task

Novices’ General Learning Effect. To determine whether the partner’s expertise affected novices’ overall reaction time improvements during the joint task, we analyzed their reaction times across the learning phase (Blocks 1 to 6) using a linear mixed-effects model (see Figure 2B). The analysis yielded a significant main effect of learning phase (*χ*^2^(1) = 85.56, *p* < 0.001, *R*^2^ = 0.25) and a significant two-way interaction between partner expertise and learning phase (*χ*^2^(1) = 12.67, *p* < 0.001, *R*^2^ = 0.04). Simple-effect follow-up tests indicated that novices paired with experts (*χ*^2^(1) = 78.92, *p* < 0.001, *R*^2^ = 0.36) demonstrated a significantly steeper rate of reaction time decline compared to those paired with pseudo-experts (χ^2^(1) = 19.41, *p* < 0.001, *R*^2^ = 0.12).

Novices’ Sequence Modulating Effect. To determine whether novices’ sequence-specific learning was actively modulated by their partner’s expertise, we analyzed their reaction time trajectories across the modulating phase (Blocks 6, 7, and 8) by comparing linear and quadratic mixed-effects models (see Figure 2B). The model comparison revealed that the quadratic model (AIC = 1603) fit the data significantly better than the linear model (AIC = 1606; *χ*^2^ = 6.92, *p* = 0.031) and showed a significant interaction between partner expertise and the squared phase term (*χ*^2^(1) = 4.39, *p* = 0.036, *R*^2^ = 0.04). Specifically, the reaction time trajectory for novices paired with trained experts followed a significant quadratic curve (*χ*^2^(1) = 8.46, *p* = 0.004, *R*^2^ = 0.15), whereas the trajectory for those paired with pseudo-experts did not (*χ*^2^(1) = 0.16, *p* = 0.690, *R*^2^ = 0.01). The quadratic model isolates sequence-specific learning from general practice by capturing the characteristic RT increase in Block 7 and subsequent recovery in Block 8.

Critically, directly contrasting reaction times in Block 6 and Block 7 showed significant main effects of block (*χ*^2^(1) = 11.34, *p* < 0.001, *R*^2^ = 0.08) and partner expertise (*χ*^2^(1) = 6.57, *p* = 0.010, *R*^2^ = 0.12), alongside a significant block-by-partner expertise interaction (*χ*^2^(1) = 8.72, *p* = 0.003, *R*^2^ = 0.14). Post-hoc comparisons confirmed that reaction times increased significantly from Block 6 to Block 7 exclusively for novices paired with trained experts. Parallel analyses for the recovery effect (Block 7 vs. Block 8) showed no significant main effects or interactions (*χ*^2^s < 2.71, *p*s > 0.103). Taken together, these findings indicate that partnering with an established expert is crucial to drive sequence-specific learning in novices, as evidenced by a pronounced interference effect in this group.

## 3. Experiment 2

Experiment 1 demonstrated that pairing with trained experts enhances implicit joint statistical learning in novices. To elucidate the mechanism driving this enhancement, we investigated whether novices rely on the transmission of nonverbal sensorimotor signals from their expert partners. Experiment 2 manipulated the availability of the partner’s action feedback, operationalized as the visual disappearance of the target following the expert’s response. By isolating these observable consequences, we tested whether immediate sensorimotor feedback is a necessary mechanism for the expert-driven learning advantage. We hypothesized that removing immediate action feedback would eliminate the novice’s sequence-specific interference effect.

### 3.1. Methods

#### 3.1.1. Participants

The target sample size was determined via an a priori power analysis using the identical parameters established in Experiment 1. To establish a standardized baseline and effectively minimize inter-cohort variance, the 56 participants (28 expert-novice dyads) from Experiment 1 were retained as the control condition (i.e., the feedback-present condition) for Experiment 2. Given that joint action effects are typically confounded by dyad-specific baseline variance, utilizing the identical control condition across experiments minimized the extraneous variance that could otherwise mask the experimental modulation. For the feedback-absent condition, we recruited an additional 56 naïve female undergraduate students (*M* = 20.63 years, *SD* = 1.62) to form 28 independent expert–novice dyads. All participants had normal or corrected-to-normal vision, provided written informed consent prior to the experiment, and received monetary compensation for their participation. The study was approved by the Institutional Review Board of Zhejiang Normal University.

#### 3.1.2. Apparatus and Stimuli

The experimental paradigm followed the overall procedure established in Experiment 1, introducing a critical manipulation during the joint statistical learning task: the availability of the expert’s action feedback to the novice. In the feedback-present condition (retained from Experiment 1), the target stimulus disappeared immediately upon the expert’s keypress, providing the novice with the spatiotemporal action dynamics of their partner’s response. Conversely, in the feedback-absent condition, the target remained on the screen for its full 1000-ms duration, regardless of the expert’s action. As in Experiment 1, structured debriefings confirmed that no participants detected the underlying sequence, indicating that all observed learning was implicit.

### 3.2. Results

#### 3.2.1. Expert Training Manipulation Validity Test

**Figure 3:**
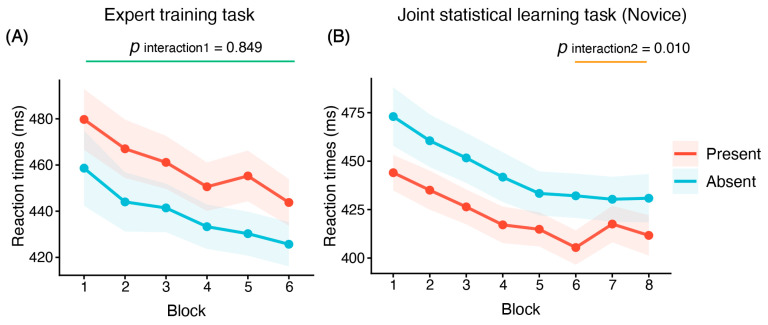
Reaction times across blocks depending on feedback availability. (**A**) Expert training task. Mean reaction times (ms) across six training blocks. (**B**) Novice performance in the joint statistical learning task. Mean reaction times (ms) across eight blocks. The green line denotes an interaction between feedback availability and learning phase (interaction1), representing the initial linear learning trend. The orange line indicates a significant interaction between feedback availability and the squared phase term during the modulating phase (interaction2), reflecting the non-linear performance changes in the latter stage. Shaded areas represent the 95% confidence intervals.

We next examined whether experts in both conditions successfully acquired the sequence regularities prior to the joint task. A linear mixed-effects model on experts’ reaction times across the training phase (Blocks 1 to 6) revealed a significant main effect of training phase (*χ*^2^(1) = 27.58, *p* < 0.001, *R*^2^ = 0.14, see Figure 3A). Crucially, neither the main effect of feedback availability (*χ*^2^(1) = 1.28, *p* = 0.257, *R*^2^ = 0.03) nor the interaction between feedback availability and training phase (*χ*^2^(1) = 0.04, *p* = 0.849, *R*^2^ = 0.01) reached significance. This confirms that experts exhibited comparable levels of sequence familiarity regardless of their assigned feedback condition.

#### 3.2.2. Novice Performance in the Joint Statistical Learning Task

Novices’ General Learning Effect. To determine whether the availability of action feedback affected novices’ overall reaction time improvements during the joint task, we analyzed their reaction times across the learning phase (Blocks 1 to 6) using a linear mixed-effects model (see Figure 3B). The analysis revealed a significant main effect of learning phase (*χ*^2^(1) = 86.19, *p* < 0.001, *R*^2^ = 0.25). Crucially, there was no significant main effect of feedback availability, nor was there a significant interaction between feedback availability and learning phase (*χ*^2^s < 3.22, *p*s > 0.103). Thus, the availability of immediate sensorimotor feedback did not modulate the novices’ general sequence acquisition during the initial learning phase.

Novices’ Sequence Modulating Effect. Corroborating with Experiment 1, a comparison between linear and quadratic mixed-effects models on novices’ reaction time trajectories across the modulating phase (Blocks 6, 7, and 8) revealed that the quadratic model (AIC = 1807) fit the data significantly better than the linear model (AIC = 1814; *χ*^2^ = 6.61, *p* = 0.037, see Figure 3B) and showed a significant interaction between feedback availability and the squared phase term (*χ*^2^(1) = 6.52, *p* = 0.010, *R*^2^ = 0.03). Specifically, the reaction time trajectory for novices in the feedback-present condition followed a significant quadratic curve (*χ*^2^(1) = 10.86, *p* < 0.001, *R*^2^ = 0.15), whereas the trajectory for those in the feedback-absent condition did not (*χ*^2^(1) = 0.16, *p* = 0.690, *R*^2^ = 0.01). Directly contrasting reaction times in Block 6 and Block 7 showed significant main effects of block (*χ*^2^(1) = 16.35, *p* < 0.001, *R*^2^ = 0.09) and feedback availability (*χ*^2^(1) = 12.49, *p* < 0.001, *R*^2^ = 0.12), alongside a significant block-by-feedback-availability interaction (*χ*^2^(1) = 10.75, *p* = 0.001, *R*^2^ = 0.15). Post-hoc comparisons confirmed that reaction times increased significantly from Block 6 to Block 7 exclusively for novices in the feedback-present condition. Parallel analyses for the recovery effect (Block 7 vs. Block 8) showed no significant main effects or interactions (*χ*^2^s < 3.27, *p*s > 0.071).

## 4. Experiment 3

Experiment 2 established that the observable consequences of a partner’s actions are necessary for enhancing novices’ implicit joint statistical learning. To further clarify this underlying mechanism, Experiment 3 investigated whether novices rely on observing the expert’s physical movements. Specifically, we manipulated novices’ perceptual access to the partner’s motor actions (i.e., the visibility of their keypresses). We hypothesized that restricting action visibility would not disrupt the novice’s sequence-specific learning, provided that immediate action feedback remained available.

### 4.1. Methods

#### 4.1.1. Participants

The target sample size for Experiment 3 was determined via an a priori power analysis, employing the identical effect size parameters as those used in Experiment 1. To isolate the effect of action visibility on learning outcomes while holding constant dyad-specific interaction styles and task familiarity, we retained the 56 participants (28 expert–novice dyads) from Experiment 1 to serve as the action-present condition. For the action-absent condition, we recruited an additional 56 naïve participants to form 28 independent expert–novice dyads. These newly recruited participants were carefully matched to the retained sample in terms of gender (all female), age (*M* = 20.54 years, *SD* = 1.95), and educational background, thereby minimizing inter-cohort variance except for the manipulated action-visibility factor. All participants had normal or corrected-to-normal vision, provided written informed consent prior to the experiment, and received monetary compensation for their participation. The study was approved by the Institutional Review Board of Zhejiang Normal University.

#### 4.1.2. Apparatus and Stimuli

The experimental paradigm followed the overall procedure established in Experiment 1, introducing a critical manipulation during the joint statistical learning task: the availability of the partner’s physical action cues. During this task, we manipulated the novice’s perceptual access to the expert’s direct motor actions. In the action-present condition (retained from Experiment 1), novices had full perceptual access to their partner’s physical movements, including the visibility of their hand movements and the audibility of their key presses. Conversely, in the action-absent condition, we occluded these direct physical cues by concealing the expert’s keyboard beneath a custom cardboard enclosure. Crucially, the observable task outcomes remained identical across both conditions; the target stimulus disappeared immediately upon the expert’s response.

As in Experiment 1, structured debriefings confirmed that no participants detected the underlying sequence, indicating that all observed learning was implicit.

### 4.2. Results

#### 4.2.1. Expert Training Manipulation Validity Test

As in Experiment 2, a significant main effect of training phase emerged (*χ*^2^(1) = 28.43, *p* < 0.001, *R*^2^ = 0.20), reflecting a general decrease in reaction times across blocks. However, neither the main effect of action visibility (*χ*^2^(1) = 0.28, *p* = 0.595, *R*^2^ = 0.01) nor the action-visibility-by-training-phase interaction (*χ*^2^(1) = 2.12, *p* = 0.146, *R*^2^ = 0.01) reached significance. This confirms that, consistent with previous experiments, experts exhibited comparable levels of sequence familiarity regardless of action visibility, establishing a balanced baseline for the joint task.

#### 4.2.2. Novice Performance in the Joint Statistical Learning Task

Novice Learning Effect. To determine whether the visibility of the expert’s actions affected novices’ overall reaction time improvements during the joint task, we analyzed their reaction times across the learning phase (Blocks 1 to 6) using a linear mixed-effects model. The analysis revealed a significant main effect of learning phase (*χ*^2^(1) = 44.73, *p* < 0.001, *R*^2^ = 0.21). Crucially, there was no significant main effect of action visibility, nor was there a significant interaction between action visibility and learning phase (*χ*^2^s < 1.42, *p*s > 0.233).

Novice Modulating Effect. A comparison between linear and quadratic mixed-effects models on novices’ reaction time trajectories across the modulating phase (Blocks 6, 7, and 8) revealed that the quadratic model (AIC = 1780) fit the data significantly better than the linear model (AIC = 1795; *χ*^2^ = 19.55, *p* < 0.001). The quadratic model revealed significant linear (*χ*^2^(1) = 11.98, *p* < 0.001, *R*^2^ = 0.14) and quadratic (*χ*^2^(1) = 11.32, *p* < 0.001, *R*^2^ = 0.14) trends for the modulating phase. Notably, no other main effects or interactions involving action visibility were significant (*χ*^2^s < 0.25, *p*s > 0.616). Taken together, these findings indicate that perceptual access to a partner’s physical movements is not necessary to drive sequence-specific learning in novices.

## 5. Discussion

The present study investigated whether novices integrate complementary statistical regularities from a trained expert partner to facilitate their joint statistical learning. In Experiment 1, novices paired with experts showed steeper declines in reaction time and a more pronounced quadratic trajectory compared to those paired with pseudo-experts, indicating a specific sensitivity to the underlying sequence rules. Experiments 2 and 3 disentangled the contributions of action visibility and action feedback to this learning advantage. Experiment 2 demonstrated that while general motor acceleration occurred regardless of feedback timing, the implicit acquisition of sequence rules required immediate action feedback. Experiment 3 further showed that restricting direct visual access to the expert’s physical movements did not attenuate the general learning advantage or the quadratic trajectory. These findings indicate that novices implicitly acquired sequence regularities by monitoring the immediate environmental outcomes of a partner’s actions rather than by observing physical kinematics.

While previous joint learning research has primarily focused on homogeneous dyads (Zheng & Wang, 2023, 2024a), the present findings demonstrate that an asymmetry in implicit sequence familiarity significantly alters joint statistical learning dynamics. Consistent with our hypothesis, Experiment 1 showed that participants paired with an expert exhibited significantly steeper declines in reaction time than those paired with a pseudo-expert, confirming that a partner’s expertise facilitates novice learning. Beyond its statistical significance, the magnitude of this effect indicates that a partner’s expertise confers a robust behavioral benefit for novices, reflecting meaningful sensorimotor facilitation in the joint task. The marginal *R*^2^ values associated with our fixed effects (ranging from 0.03 to 0.15) may appear modest. This pattern is theoretically expected. First, implicit sequence learning manifests as subtle RT modulations embedded within a larger general practice effect, which is particularly pronounced in joint action contexts and absorbs substantial variance. Second, our LMMs include random intercepts for dyads and individuals, partitioning out baseline variability. The marginal *R*^2^ isolates variance uniquely attributable to fixed manipulations, whereas the conditional *R*^2^ is considerably larger. Thus, a modest marginal *R*^2^ does not indicate a weak phenomenon, but rather reflects a controlled model that has successfully stripped away confounding interpersonal noise. This facilitative effect aligns with research in decision-making, motor coordination, and musical performance, domains in which expertise-driven advantages are well-documented (Schultz & Palmer, 2019; Sella et al., 2018; van Nooijen et al., 2025; Wolf et al., 2018). Notably, unlike studies involving explicit pedagogical roles, the current paradigm produced facilitation under implicit conditions where even the experts lacked conscious awareness of the sequence rules. Although real-world experts often possess explicit knowledge (e.g., a teacher explicitly explaining a concept), a substantial portion of human knowledge transfer actually relies on implicit expertise. For example, an infant implicitly acquires grammatical regularities from a parent’s natural speech (Tamis-LeMonda et al., 2014), and a novice dancer unconsciously adapts to an experienced partner’s rhythm without any verbal instruction (Chang et al., 2020). By operationalizing expertise as implicit sequence familiarity, our paradigm isolates the foundational sensorimotor mechanisms underlying the implicit transmission of task regularities. This modeling highlights that, even in the absence of explicit pedagogical intent or conscious awareness, an expert’s stable behavioral predictions alone can serve as a statistical scaffold for novice learning (van Nooijen et al., 2024).

To isolate the specific sensorimotor cues driving this effect, Experiment 2 tested whether the immediate perceptual consequences of a partner’s action underpin joint learning facilitation. Although previous research indicates that representing a partner’s perceptual feedback is essential for real-time motor coordination (Curioni et al., 2019; Pfordresher, 2006; Shea et al., 2016), our results extend this principle to the domain of cognitive sequence acquisition. Without immediate visual feedback, novices failed to exhibit the quadratic reaction time trajectory that characterizes the acquisition of statistical rules. This requirement for immediate feedback provides empirical support for the Theory of Event Coding, which posits that actions are cognitively represented through their anticipated perceptual effects (Dolk et al., 2013; Hommel, 2019). In the joint task, novices exhibit sequence-specific behavioral adaptation by adjusting their responses to the immediate visual offset of the target following the partner’s response (Obhi & Hall, 2011; Varga et al., 2024; Vogel et al., 2021). By observing the exact timing of this stimulus offset, novices implicitly infer the expert’s response speed and their underlying familiarity with the sequence rules. Without these immediate sensory consequences, novices are fundamentally unable to generate a reliable internal model of the expert’s task regularities. This interpersonal action-binding mechanism suggests that novices implicitly extract and internalize the partner’s task regularities by merging their own motor plans with the anticipated sensory outcomes generated by the partner, effectively bypassing the need to encode the expert’s specific motor kinematics (Buehner, 2012). Ultimately, this demonstrates that epistemic asymmetry facilitates joint learning precisely because novices can seamlessly incorporate the expert’s statistical regularities through shared perceptual effects. These findings significantly refine previous joint action models (e.g., Bolt & Loehr, 2017; Sebanz et al., 2006) by revealing that shared predictive representations are anchored in shared sensory outcomes rather than mere physical co-presence. Alternatively, one might argue that novices’ performance gains reflect generalized rhythmic entrainment rather than the acquisition of statistical regularities. However, our findings argue against this purely rhythmic account. If facilitation were driven by rhythmic entrainment, performance benefits would remain invariant across task conditions. In contrast, the specific interference effects in Experiment 1 and the critical reliance on immediate action feedback in Experiment 2 demonstrate that novices were not passively entrained; instead, they were actively integrating sequence-specific regularities, confirming that the observed facilitation is driven by rule-based learning rather than generalized temporal benefits.

Experiment 3 investigated the necessity of direct visual access to a partner’s physical movements for the exchange of nonverbal information. The results indicated that the novices’ statistical learning remained robust even when they could not see the expert’s physical actions. Interestingly, this finding diverges from prior evidence highlighting the critical role of shared visual information in joint coordination (Lokesh et al., 2022; Vesper et al., 2016b). This discrepancy likely arises from fundamental differences in task demands. Studies demonstrating the necessity of action visibility typically involve continuous spatial coupling or synchronous execution, where tracking a partner’s specific kinematics is essential. In contrast, joint statistical learning is primarily a cognitive acquisition process anchored in shared perceptual outcomes. As established in Experiment 2, novices do not need to encode the expert’s exact physical movements; rather, they rely on the immediate sensory consequences of the partner’s actions. When direct vision is restricted, the expert partner conveys sequence regularities through alternative nonverbal channels, such as predictable temporal dynamics. Consequently, novices can recruit internal action simulations to predict partner behavior and maintain shared representations without the need for continuous visual observation.

Several constraints of the current experimental design merit consideration when interpreting these findings. First, the gender homogeneity of our sample warrants consideration; our dyads were exclusively female to control for extraneous variance associated with mixed-gender social dynamics. Consequently, the generalizability of these results to male-male or mixed-gender dyads remain to be established. Second, the current paradigm employs a highly controlled joint serial reaction time task. While this controlled setting was necessary to isolate the underlying implicit learning mechanisms, it may limit the ecological validity of the findings. Real-world asymmetric joint actions—such as sports or musical ensembles—typically involve continuous, dynamic, and multi-modal interactions. Therefore, future research is needed to determine whether these implicit learning mechanisms hold in more ecologically valid, naturalistic environments. Third, the reuse of Experiment 1 participants as the control cohort for Experiments 2 and 3 may introduce inter-cohort variability due to differences in recruitment timing. Although this design choice was implemented to minimize dyad-specific variance and optimize statistical power, we acknowledge the potential for cohort effects, and future replications with independent control cohorts in each experiment would help establish the robustness of the current findings.

## 6. Conclusions

Grounded in foundational joint action models, previous studies have established that interacting individuals can form shared statistical representations during homogeneous interactions. However, little attention has been paid to how e asymmetries in sequence familiarity influence joint learning dynamics, particularly when novices need to extract statistical regularities from a pre-trained partner without explicit instruction. Furthermore, prior research has not sufficiently disentangled the specific nonverbal sensorimotor channels (i.e., action visibility vs. action feedback) through which these implicit regularities are conveyed interpersonally. The present study addressed these gaps by implementing a joint serial reaction time paradigm. Crucially, rather than suggesting a general mechanism of explicit knowledge transfer, our data revealed a specific behavioral phenomenon: novices implicitly extract and integrate sequence-related regularity from a more knowledgeable partner’s nonverbal cue. Moreover, our experimental manipulations revealed that immediate perceptual action feedback, rather than direct visual access to the expert’s physical movements, is fundamentally necessary to drive this learning advantage.

Theoretical and practical implications derived from these findings must be interpreted cautiously within the boundaries of our experimental design. Theoretically, these findings extend current models of joint statistical learning and provide empirical support for the Theory of Event Coding within asymmetric contexts, demonstrating that shared predictive representations are constructed through anticipated sensory outcomes rather than shared physical kinematics. Practically, our contributions highlight the mechanisms of implicit, nonverbal behavioral facilitation. Ultimately, this work establishes that sensorimotor integration alone is sufficient to drive sequence-specific learning in expert-novice dyads, avoiding overgeneralizations to explicit pedagogical environments.

## Data Availability

All the data and procedure are available on the Open Science Framework (https://osf.io/p2458/) (accessed on 18 May 2026).

## Abbreviation

The following abbreviation is used in this manuscript:

TEC	Theory of Event Coding
